# NK Cells: Not Just Followers But Also Initiators of Chronic Vascular Rejection

**DOI:** 10.3389/ti.2024.13318

**Published:** 2024-10-16

**Authors:** Mathilde Chambon, Alice Koenig

**Affiliations:** ^1^ CIRI, INSERM U1111, Université Claude Bernard Lyon I, CNRS UMR5308, Ecole Normale Supérieure de Lyon, University of Lyon, Lyon, France; ^2^ Hospices Civils de Lyon, Edouard Herriot Hospital, Department of Transplantation, Nephrology and Clinical Immunology, Lyon, France; ^3^ Lyon-Est Medical Faculty, Claude Bernard University (Lyon 1), Lyon, France

**Keywords:** antibody mediated rejection, chronic rejection, natural killer cells, missing self, antibody dependent cellular cytotoxicity

## Abstract

Chronic graft rejection represents a significant threat to long-term graft survival. Early diagnosis, understanding of the immunological mechanisms and appropriate therapeutic management are essential to improve graft survival and quality of life for transplant patients. Knowing which immune cells are responsible for chronic vascular rejection would allow us to provide effective and appropriate treatment for these patients. It is now widely accepted that natural killer (NK) cells play an important role in chronic vascular rejection. They can either initiate chronic vascular rejection by recognizing missing self on the graft or be recruited by donor-specific antibodies to destroy the graft during antibody-mediated rejection. Whatever the mechanisms of activation of NK cells, they need to be primed to become fully activated and damaging to the graft. A better understanding of the signaling pathways involved in NK cell priming and activation would pave the way for the development of new therapeutic strategies to cure chronic vascular rejection. This review examines the critical role of NK cells in the complex context of chronic vascular rejection.

## Introduction

Solid organ transplantation represents the optimal treatment option for patients with end-stage organ failure [1]. Nevertheless, the survival of allografts is constrained by the occurrence of rejection, which is initiated when the recipient’s immune system identifies donor determinants [in particular, mismatched Human Leucocyte Antigen (HLA) molecules] and conducts the destruction of the graft. Over the past few decades, the development of immunosuppressants has enabled significant improvements in short-term graft survival, largely by preventing early rejections, which are mainly driven by T cells. However, the percentage of grafts that do experience attrition beyond the first transplant year remains unchanged [2]. Long-term graft survival represents an unmet medical need in solid organ transplantation. In the majority of cases, long-term graft loss is of immunological origin and is referred to as chronic rejection. Although there are discrepancies in the lesions found in cases of chronic rejection in the different organs, it is now clear that vascular lesions are a common feature [3]. It is widely accepted that these lesions may be the result of mechanisms involving donor specific antibodies (DSA) [3]. However, in some cases, antibodies are not present, suggesting that other mechanisms may also be involved in chronic vascular rejection. Recent research has challenged traditional views on chronic vascular rejection, revealing a prominent role for Natural Killer (NK) cells in chronic vascular rejection, regardless of the presence or absence of DSA. This challenges the prevailing view that innate immune cells only play a role in rejection if they are recruited by the adaptive immune system. The objective of this review is to examine the role of NK cells in chronic vascular rejection, with a particular focus on the mechanisms that can trigger their activation. This will provide insights into the pathways involved and identify potential therapeutic avenues for preventing their activation and, consequently, reducing the occurrence of chronic vascular rejection.

## General Overview of NK Cell Activation

**FIGURE 1:**
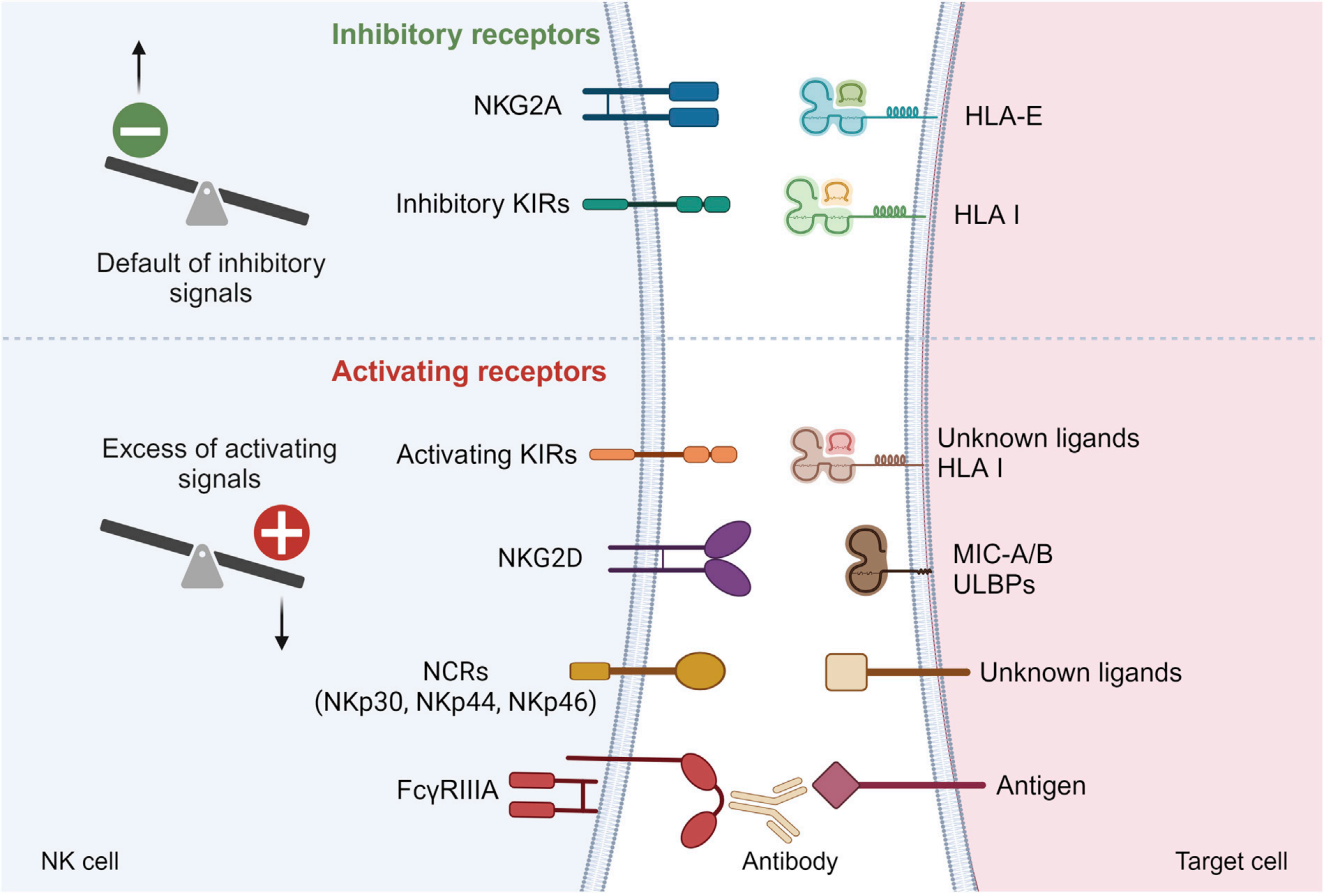
Main inhibitory and activating receptors on NK cell surface. Default of inhibitory signals or excess of activating signals can lead to NK cell activation. KIRs, Killer Immunoglobin-like Receptors; NCRs, Natural Cytotoxicity Receptors; HLA, Human Leucocyte Antigen; MIC-A/B, MHC class I-related chain A/B; ULBPs, UL16-binding proteins.

NK cells are a crucial component of the innate immune system, responsible for detecting and eliminating infected, stressed, or transformed cells, such as cancerous cells. Unlike T cells and B cells, which are part of the adaptive immune system and require prior exposure to a specific pathogen for an effective response, NK cells can respond immediately to threats without prior sensitization. To achieve this, NK cells express several germline-encoded activating and inhibitory receptors on their surface (Figure 1). The balance between these signals determines whether the NK cell will attack the target cell. NK cells can be activated through three main mechanisms. First, missing-self recognition occurs when a target cell loses or downregulates its HLA class I molecules, often due to viral infection or cancer (Figure 1). The lack of inhibitory signals allows NK cells to destroy the target cell. Second, NK cells can be activated by induced-self ligands. Cells under stress or transformation may express specific ligands, such as MICA/B or ULBPs, which are recognized by activating receptors on NK cells, triggering NK cell activation (Figure 1). Finally, NK cells can be activated through antibody-dependent cellular cytotoxicity (ADCC). In this process, NK cells recognize antibodies bound to the surface of target cells via their Fcγ receptor (FcγRIIIA) (Figure 1). This antibody-mediated recognition leads to NK cell activation and targeted killing of the antibody-coated cell.

In the following sections of the review, we will describe how these mechanisms of NK cell activation might be involved in chronic vascular rejection.

## Missing-Self Induced NK Cell Activation and “Innate” Rejections

### Inhibitory Killer Cell Immunoglobulin-Like Receptors (KIR) Bind to HLA Class I

Inhibitory KIRs are highly polymorphic at the genetic level, heterogeneously expressed by NK cells. Inhibitory KIRs possess two (2D) or three (3D) extracellular immunoglobulin domains, a transmembrane domain and a long (L) cytoplasmic tail containing two immunoreceptor tyrosine-based inhibitory motifs (ITIMs) [4]. Each inhibitory KIR has for ligands a subgroup of HLA class I allotypes. Inhibitory KIRs with 2 extracellular domains recognize HLA-C molecules based on two dimorphisms at position 77 and 80 of the α1 domain of the α chain [4]. KIR2DL1 associates with HLA C2 allotypes [4]. In contrast, KIR2DL2 and three show specificity for HLA C1 allotypes [4]. KIR3DL1 recognizes HLA-A and HLA-B molecules that display the Bw4 motif [5]. Finally, KIR3DL2 solely interacts with HLA-A3 and A11 molecules [4].

### NK Cell Education and Activation by Missing-Self

**FIGURE 2:**
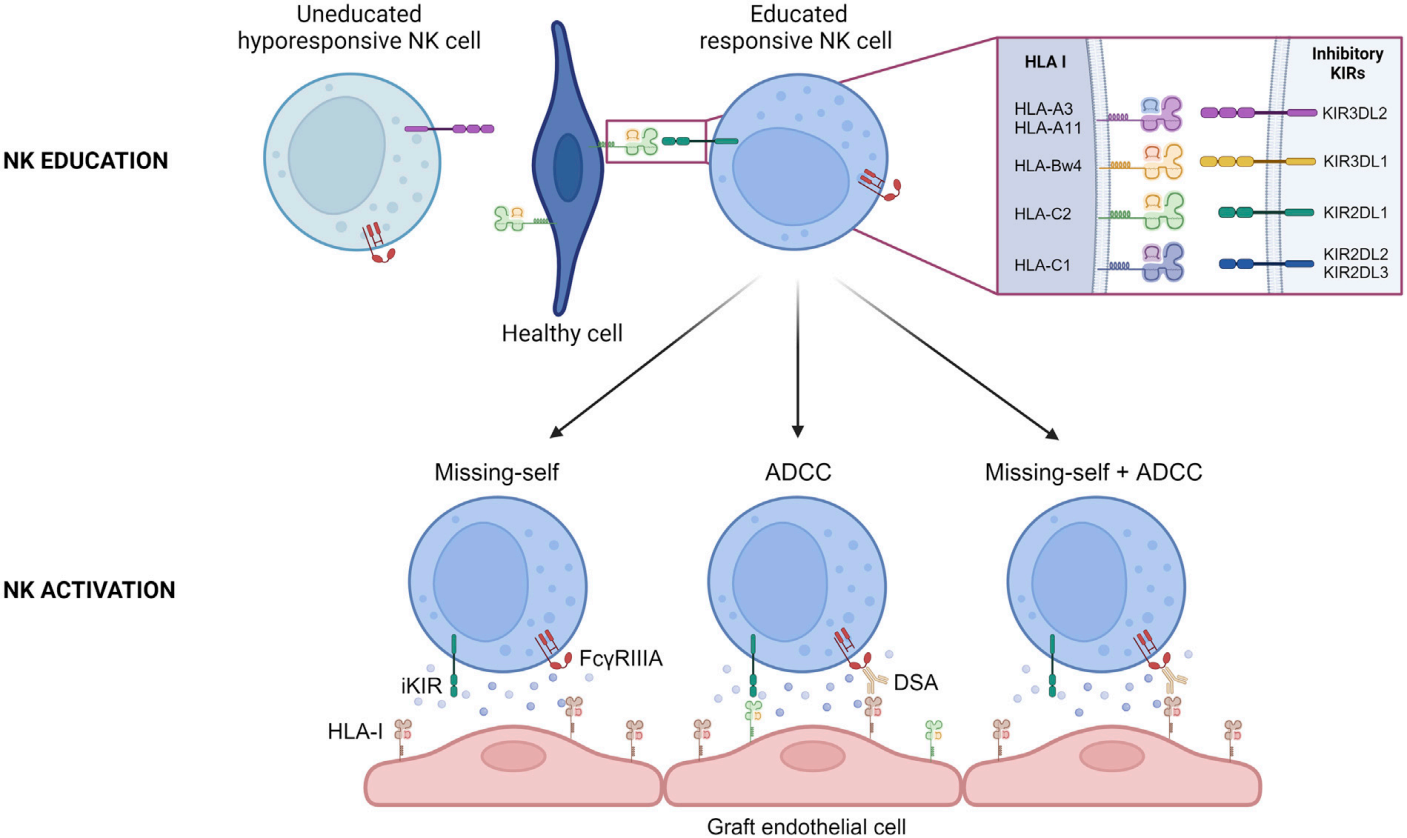
NK cell education and activation during chronic vascular rejection. Schematic representation of the education process of NK cells during their maturation. The interaction between specific inhibitory KIRs on NK cell surface and HLA I on healthy cells allows NK cell to become responsive. Schematic representation of the three situations that can conduct to recipient NK cell activation during chronic vascular rejection. ADCC, Antibody Dependent Cell Cytotoxicity; DSA, Donor Specific Antibody; HLA-I, Human Leucocyte Antigen class I.

The complex interplay between inhibitory KIRs and HLA class I molecules is of paramount importance in the education and activation of NK cells. As the genes for inhibitory KIRs and HLA class I molecules are on different chromosomes and that NK cells express germline-encoded receptors that do not undergo somatic rearrangement during development, NK cells need to undergo an educational process where they learn to distinguish “self” from “non-self” through interactions between inhibitory KIRs and self HLA class I molecules [6]. NK cells that encounter self-HLA class I molecules during their maturation process receive inhibitory signals, ensuring that they do not attack healthy cells displaying self HLA class I molecules (Figure 2). In absence of any interaction between inhibitory KIRs and HLA class I molecules during their development, NK cells become hypo responsive (Figure 2). This process helps to refine NK cell self-tolerance and future reactivity as the number of interactions between inhibitory KIRs on NK cells and HLA class I molecules determines the degree of responsiveness of mature NK cells. Conversely, when these educated NK cells encounter virally infected or transformed cells that lack or display altered HLA class I molecules, the lack of inhibitory signals transmitted by inhibitory KIRs allows for the activation of NK cells by the recognition of missing self (Figure 2).

### Missing-Self Induced NK Cell Activation Triggers Rejection After Organ Transplantation

For a considerable period, the implication of the “missing self” in organ transplantation was overlooked. New advances were made when Colvin and his team observed that when a heart transplant from a parental strain was transplanted to an F1 hybrid strain (between the parental line and a second strain), the graft presented chronic vascular rejection [7]. It is notable that this model demonstrates that NK cells can initiate chronic vascular rejection, but that they are insufficient to trigger chronic vascular lesions when acting alone. For this to occur, they must recruit T cells that are not specific for donor major histocompatibility complex (MHC) and macrophages in particular, through the secretion of interferon gamma (IFN-γ) [7, 8]. In humans, many studies have looked at this issue for a long time without results, probably because of a lack of data to analyze the presence of missing-self correctly [9–13], or because the chosen read out was not the good one [10], or because of confounding factors [14, 15]. A decade ago, in a cohort of 137 kidney transplant patients compatible for HLA-A, HLA-B and HLA-DR (i.e., in whom minimal T- and B cell alloreactivity was expected), Van Bergen et al. observed that missing self was associated with a worse allograft survival [14]. This was one of the first clinical studies to suggest the involvement of missing self after kidney transplantation, but to go deeper into the demonstration, we have to wait for a study published by our group a few years ago [15]. In a cohort of deeply phenotyped kidney transplant patients, we demonstrated that a greater proportion of patients with microvascular inflammation lesions in the absence of DSA (either HLA or non-HLA DSA) exhibited a missing self on their graft susceptible to being sensed by the recipient’s NK cells and this resulted in reduced allograft survival compared to a control cohort (patients with no vascular lesions and no DSA) [15]. This clinical correlation was confirmed in an *in vitro* model in which we demonstrated that the lack of self HLA class I molecules on endothelial cells can activate NK cells, which in turn induce endothelial cell damage (Figure 2) [15]. An *in vivo* mouse model of heart transplantation was then developed. Heart transplants were performed from β2 micro KO mice (i.e., mice that do not express MHC class I molecules) to mice of the same genetic background [15]. The grafts developed microvascular inflammation lesions only in the presence of NK cells and a missing self, thereby confirming the existence of missing-self induced NK-mediated rejection [15]. Our results were confirmed in a population-based study which showed that missing self increased microvascular inflammation occurrence after kidney transplantation, independently of DSA [16]. Microvascular inflammation severity increased with the number of missing self [16].

## NK Cell-Mediated ADCC During B Cell-Mediated Rejections

### Key Role of NK Cells During Chronic Antibody-Mediated Rejection (AMR)

It is widely accepted that NK cells play a pivotal role in the development of chronic vascular rejection in the presence of DSA. Binding of circulating DSA to directly accessible donor HLA molecules expressed by graft endothelial cells can sometimes trigger the classical complement pathway, which accelerates the rejection process [17, 18]. However, this is not a mandatory requirement for the development of chronic AMR [19]. It has been demonstrated that the recruitment of innate immune cells by DSA is sufficient to trigger endothelial cell damage through an ADCC mechanism (Figure 2). In 2012, Colvin and his team have shown that when RAG^−/−^C3^−/−^ mice were transplanted with an allogeneic heart and injected regularly with DSA, they developed chronic vascular lesions in their graft and that these lesions were completely abrogated in the absence of NK cells [20]. In the clinical setting, the key role of NK cells in AMR is supported by transcriptomic analyses of renal graft biopsies [21–23]. Recent data suggesting that afucosylation of HLA-specific IgG1 is directly related to antibody pathogenicity in kidney transplantation also provides indirect evidence for the key role of NK cells in AMR, as fucosylation is known to strongly influence the affinity of IgG for FcγRIIIA, which is expressed by NK cells [24, 25]. Furthermore, the intensity of NK cell infiltration within the graft correlates with graft survival after kidney transplantation [26].

NK cell activation during AMR is thought to be triggered by the interaction between NK’s unique Fcγ receptor, FcγRIIIA and DSA (Figure 2). In the murine heart transplantation model of AMR described above, mice injected with F (ab′)_2_ DSA fragments failed to develop chronic vascular lesions in comparison to mice injected with intact DSA, suggesting that the interaction of NK cells with the Fc fragment of DSA is necessary [20]. Transcriptomic analyses conducted on sets of renal allograft biopsies from patients with AMR identified NK cell signaling, including evidence for the FcγRIIIA signaling elements [27, 28] suggestive of an NK cell activation through FcγRIIIA in AMR. In a recent study, thanks to single cell RNA sequencing analyses and multiplexed immunofluorescence, Lamarthée and colleagues have shown an association between FcγRIIIA+ NK cells and the severity of intragraft inflammation in the context of AMR [22]. Like in missing-self induced NK-mediated rejections, NK cells do not act alone and seem to interact in particular with FcγRIIIA+ non-classical monocytes via LGALS9-HAVCR2 to trigger allograft destruction [22]. They also interact to a lesser extent with T cells expressing CD74CXCR4 via the secretion of macrophage migration inhibitory factor (MIF) [22].

### Factors Modulating NK Cell Activation During AMR

A single nucleotide polymorphism exists in the FcGR3A gene, resulting in the expression of two co-dominant alleles coding for either a phenylalanine (F) or a valine (V) at amino acid position 158 in the extracellular domain of the receptor [29]. This single nucleotide polymorphism modulates the binding capacity of FcγRIIIA to the Fc fragment of IgG, which may in turn modulate the severity of lesions and the prognosis of patients with AMR. In kidney transplantation, Arnold et al. observed that patients with DSA and presenting at least one high binding allele (V/V or V/F) exhibited more frequent and severe peritubular capillaritis lesions [30]. Furthermore, the researchers hypothesize that this heightened microvascular inflammation may be due to the secretion of IFN-γ, which facilitates the recruitment of other immune cells in the graft [30]. These findings were corroborated in a cohort of kidney transplant recipients with chronic humoral rejection [31]. The patients with two V/V alleles exhibited a higher glomerulitis score and a reduced graft survival compared to patients of other genotypes [31]. The authors propose that the V-allele is associated with an increased expression of FcγRIIIA by NK cells, which translates into a greater propensity for NK cells to degranulate when they interact with their targets [31].

In addition, a recent study has investigated the impact of different genetic variations present in the genes of different NK cells in a cohort of patients with *de novo* DSA [32]. In particular, they got interested in a polymorphism in *KLRC2* gene which encodes NKG2C, an activating NK cell receptor, which binds to HLA-E and is known to be present on memory like NK cells with more potent effector functions [29]. Heterozygous or homozygous *KLRC2* deletion (*KLRC2* ^del^) is associated with a significantly lower or absent NKG2C expression level. Here, they showed that patients with a *KLRC2* ^wt/wt^ genotype presented more microvascular lesions than *KLRC2* ^wt/del^ patients but failed to show any impact on allograft survival [32].

Finally, our group has recently shown that missing self and DSA can combine to induce chronic vascular rejection in a cohort of kidney transplant patients with AMR (Figure 2) [33]. Notably, the additive effect of missing self on allograft survival was only observed in patients with ADCC-dependent AMR [33]. This may be explained by the fact that allograft loss is too rapid in patients with complement-dependent AMR.

Whether the NK cell initiates rejection in missing self-induced rejection or is recruited by the adaptive immune system in AMR, it seems that a final common pathway involving NK cell is triggered during chronic vascular rejection. This was suggested in particular by a study which showed that the transcriptomic signatures were similar in biopsies of patients with DSA-positive and DSA-negative microvascular inflammation lesions [34]. These patients showed similar upregulation of pathways such as IFNγ-induced pathways and NK cell activation, and similar enrichment of infiltrating leukocytes [34]. A better understanding of the pathways leading to NK activation may therefore be useful in the development of treatments targeting NK cells in chronic vascular rejection, whether or not DSA are present.

## Signaling Pathways Involved in NK Cell Priming and Activation

To become fully activated, NK cells need to receive a priming signal from cytokines and a second signal that results from the balance of inhibitory and activating signals transmitted by their many inhibitory and activating receptors (Figure 1).

### NK Cell Priming

**FIGURE 3:**
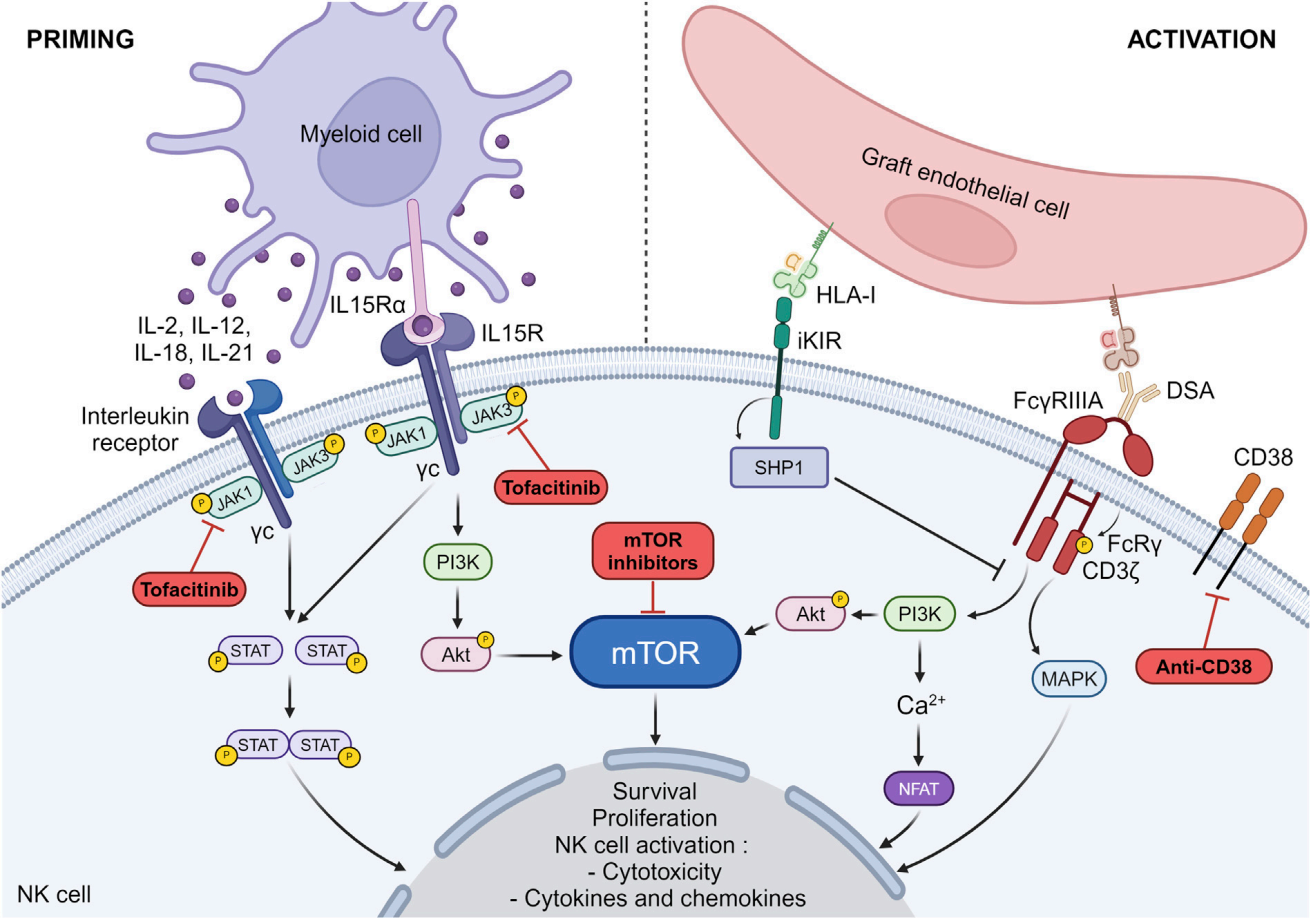
NK cell priming and activation pathways. The pathways involved in NK cell priming and activation as well as the drugs susceptible to block these pathways are depicted. IL15Rα, IL-15 receptor alpha subunit; IL15R, IL-15 receptor; DSA, Donor specific antibody; iKIR, inhibitory killer cell immunoglobulin-like receptors; MAPK, mitogen-activated protein kinase; HLA-I, Human Leucocyte Antigen class I; NFAT, nuclear factor of activated T-cells.

Among the various cytokines that may play a role in NK cell priming (IL-2, IL-12, IL-15, IL-18 and IL-21), IL-15 is of particular importance in regulating NK cell homeostasis and activation [35–37]. IL-15 binds to IL-15Rα expressed on antigen presenting cells and is then presented in trans to the IL-2/IL-15Rβγ heterodimer on NK cells. IL-15 induces the phosphorylation of JAK1/JAK3, allowing the recruitment and activation of the transcription factor STAT5 (Figure 3), which then translocates to the nucleus to support NK cell survival [38]. In parallel, it induces the activation of the PI3K-AKT-mTOR signaling pathway which promotes the acquisition of cytolytic potential by NK cells when they will encounter abnormal cells (Figure 3) [38].

In the context of transplantation, we and others have shown that NK cells need to undergo priming to get activated against the graft [15, 39, 40]. In particular, we have shown that prolonged cold ischemia (suggesting significant ischemia-reperfusion injury) and viral infections such as cytomegalovirus can lead to NK cell priming after transplantation, a prerequisite for NK cell activation and the development of chronic vascular rejection in the presence of a missing self [15]. These two types of events favor the induction of IL-15 and its trans presentation by dendritic cells, thereby promoting NK cell cycle entry, proliferation, survival and cytotoxicity [35, 41–44]. In addition, cytomegalovirus also induces a remodeling of the NK phenotype with an increase in NK cell populations expressing educated inhibitory KIRs and expression of markers such as NKG2C and CD57 [45, 46]. This subpopulation of NK cells is considered to be a memory like population and is known to be better at exhibiting cytotoxicity and secreting cytokines such as IFN-γ [46].

Finally, a recent study also showed that IL-21 may help NK cells to differentiate into polyfunctional type 1 activated cytotoxic effectors with a potentially deleterious ability to infiltrate the kidney allograft during AMR and damage the vascular endothelium [47].

### NK Cell Activation

Activating NK cell receptors are associated with different adaptor molecules which contain immunoreceptor tyrosine-based activation motifs (ITAMs) on their cytoplasmic domains. FcγRIIIa receptor which is a low affinity receptor for IgG forms a complex with 2 adaptor molecules FcRγ and CD3ζ. Upon ligation of the Fc fragment of IgG by FcγRIIIa, the ITAMs are phosphorylated. This triggers a cascade of downstream events involving the PI3K pathway, the mitogen-activated protein kinase (MAPK) pathway and calcium release conducting to the activation of nuclear factor of activated T-cells (NFAT) (Figure 1) [48–50]. Together, these pathways contribute to the cellular response leading to NK cell activation [51–53]. Unlike activating receptors, inhibitory receptors and in particular inhibitory KIRs have 2 ITIMs in their cytoplasmic tails. When inhibitory KIRs interact with their self-MHC class I ligands, the ITIMs are phosphorylated and recruit the tyrosine phosphatases such as SHP-1 (Src homology containing tyrosine phosphatase-1), which remove phosphate groups from several proteins downstream of activating receptors, thereby preventing NK cell activation (Figure 1) [54]. In the absence of ligation of their self-MHC class I ligands, inhibitory KIRs do not interfere with activating signals, allowing NK cell activation. We have shown that missing cell induced-NK cell activation triggers the mTOR pathway [15]. Whether this is also the case for FcγRIIIa-induced NK cell activation is not clear, but experimental data investigating NK cell activation by other activating receptors suggest that this is certainly the case [55].

Activation of NK cells can lead to killing of the target cell. NK cells exert their cytotoxic activity through two main pathways: the exocytosis of lytic granules containing perforin and granzymes A and B; and the expression of ligands (FAS ligand, TRAIL) of death receptors (FAS, DR4 and DR5) expressed on target cells. In addition, NK cells can secrete pro-inflammatory cytokines (TNF-α and INF-γ) that favor the activation of dendritic cells, T cells and monocytes [56]. They also secrete chemokines such as CCL-2 (MCP-1), CCL3 (MIP-1α), CCL4 (MIP-1β), CCL5 (RANTES), which attract effector lymphocytes and myeloid cells to inflamed tissues [57, 58]. In a murine heart transplantation model of AMR, Lin and colleagues showed that both IFN-γ and contact-dependent cytotoxicity are necessary for NK cells to induce chronic vascular lesions [59]. The same observations have been made in the hybrid resistance model regarding the need for IFN-γ to help NK cells recruit T cells and monocytes to induce the vascular lesions [7, 8]. Several clinical studies have shown that the transcripts involved in IFN-γ pathway and NK cell-mediated cytotoxicity are upregulated in biopsies of patients with HLA positive or negative microvascular inflammation lesions [28, 34].

## Targeting NK Cells: An Interesting Lead to Prevent and Treat Chronic Vascular Rejection?

### Prevention of NK-Driven Chronic Rejection

Transplant patients classically received a two- or three-drug regimen consisting of a calcineurin inhibitor, an antiproliferative agent, and corticosteroids. The aim of these treatments is to inhibit T-cell activation. Theoretically, calcineurin inhibitors could have an effect on NK cell activation because NFAT is expressed by NK cells [50]. In a first *in vitro* study, human NK cells were treated with cyclosporine and different cytokines (IL-2 or IL-15) for 7 days. This study confirmed that the NFAT pathway was blocked, especially in CD56^dim^CD16+inhKIR+ NK cells, and that this resulted in a decrease in NK cell proliferation but an increase in cytotoxicity against different target cells and an increased ability to secrete IFN-γ after a new stimulation with IL-12 and IL-18 [60]. Two more recent studies confirm the conserved cytotoxicity of NK cells exposed to cyclosporine or tacrolimus and activated by the two mechanisms involved in chronic rejection (MS and ADCC) [61, 62]. *In vivo*, we confirm that cyclosporine does not prevent missing-self-induced NK-mediated rejection in our murine heart transplant model, suggesting that this immunosuppressant may have a limited effect in preventing chronic vascular rejection in patients [15]. The effect of antiproliferative drugs such as azathioprine and mycophenolate mofetil and low-dose corticosteroids on NK cells has been less studied. Some *in vitro* studies suggest that these drugs may prevent NK cell activation, but these data should be treated with caution due to the lack of *in vivo* data [61, 62].

Alternatively, transplant recipients may receive mTOR inhibitors (mammalian target of rapamycin) as part of their maintenance regimen. As previously depicted, mTOR pathway plays an important role in NK cell education, priming and activation [38, 55]. *In vitro* studies have shown that mTOR inhibitors can prevent NK cell cytotoxicity [55, 61]. *In vivo*, we confirm that rapamycin can efficiently prevent missing-self induced NK-mediated rejection in our murine heart transplantation model [33]. Finally, a recent clinical study in a cohort of lung transplant recipients treated with rapamycin showed that their NK cells had reduced mTOR activity, which was associated with decreased cell proliferation and lower levels of FcRγ, the adaptor molecule of FcγRIIIa, suggesting that they may be less efficient at performing ADCC [63]. A pilot clinical trial (NCT03955172) is currently underway to assess the efficacy of mTOR inhibitors in preventing the formation of chronic lesions in kidney transplant patients presenting a missing-self induced NK-mediated rejection. Although promising, the universality of mTOR inhibitors could be questioned, as this treatment is associated with many side effects, leading to its discontinuation in 30%–50% of patients, and cannot be used late in the course of chronic rejection, as mTOR inhibitors are poorly tolerated in patients with chronic glomerular lesions, as they prevent the adaptation of podocytes to stress [64].

As the cytokine priming process is necessary for all NK cell mechanisms involved in chronic vascular rejection, blocking it offers a unique opportunity to prevent the deleterious effects of NK cells during chronic vascular rejection. As NK cell priming may be the result of multiple cytokines, rather than trying to block one cytokine, blocking the pathways which result from the binding of cytokines on their receptors which share the common γc chain may be more appropriate. Tofacitinib is an oral JAK inhibitor that selectively inhibit intracellular cytokine signaling mediated by JAK3 and/or JAK1. A recent study in a rat model of mixed cellular and antibody-mediated rejection indicates that tofacitinib effectively reduces the infiltration of T cells and NK cells into the graft, thereby limiting the progression of lesions and improving graft and recipient survival [65]. Its use in renal transplant patients has been tested in a clinical randomized phase 2b trial [66]. Patients received either tofacitinib (low or high dose) or cyclosporine in combination with mycophenolic acid and corticosteroids [66]. Tofacitinib was equivalent to cyclosporine in preventing acute rejection and was associated with an improved renal function and less tubular atrophy and interstitial fibrosis [66]. However, it was also responsible for more cancerous and infectious complications in the high dose tofacitinib regimen [66]. This side effect may be the reflect of the efficient blocking of NK cells which are well known for their role in anti-viral and anti-tumoral immunity.

### Treatment of NK-Driven Chronic Rejection

The current consensus for the treatment of AMR associates rapid depletion of circulating DSA with plasmapheresis with a combination of corticosteroids and high-dose intravenous immunoglobulins (IVIg) [67]. This costly and prolonged therapeutic approach has a reported 3-year graft survival of <50% [67]. By preventing complement activation, these treatments slow down the course of rejection and convert it from acute to chronic rejection in which ADCC is the main mechanism of graft destruction.

Few studies have investigated the effect of high-dose corticosteroids on NK cells. In one *in vitro* study, human purified NK cells and T cells were incubated with elevated doses of hydrocortisone, comparable to drug levels achieved in patients receiving 1 mg/kg to 1 g of methylprednisolone. In contrast to T cells, NK cells were resistant to steroid-induced apoptosis and their cytolytic capacity against MHC class I deficient tumoral cell was not affected [68]. In the same line, although methylprednisolone seems to affect NK cell activation through some activating receptors such as NKp46, NKG2D or 2B4, it does not seem to prevent FcγRIIIA-mediated NK cell cytotoxicity [69]. High dose IVIg may also prevent NK cell activation by saturating all FcγRIIIa receptors present on NK cells, rendering them blind to DSA. However, their impact on NK cells is still debated [67].

To achieve a sustained therapeutic effect in AMR, attempts have been made to target DSA-producing cells with either anti-CD20 monoclonal depleting antibody (rituximab) or a proteasome inhibitor (bortezomib) without efficacy [70, 71]. Recently, an antibody targeting CD38 (felzartamab), a receptor present on plasma cells and NK cells, has been tested in a randomized phase 2 trial study for the treatment of renal transplant recipients diagnosed with late active or chronic-active antibody-mediated rejection after kidney transplantation [72]. This treatment efficiently reduced rejection lesions. This improvement seems to be due to NK cell depletion rather than plasma cells depletion as DSA MFI barely decreased with the treatment. These data need to be confirmed in a larger cohort of patients. A further clinical trial is currently underway to test an alternative anti-CD38 (daratumumab) in the same indication (NCT05913596). If effective, its use can be extended to missing self-induced NK-mediated rejection.

## Conclusion

In conclusion, this review has explored the role of NK cells in chronic vascular rejection. They appear to be the masters of chronic vascular rejection, regardless of whether DSA are present or not. This makes them a prime target for attempts to prevent and/or treat chronic vascular rejection, which is currently the leading cause of allograft loss. Inhibition of NK cell priming and activation represent interesting avenues for preventing NK-mediated chronic vascular rejection, while drugs that deplete NK cells may be of interest for the treatment of chronic vascular rejection. By reducing chronic vascular rejection, these strategies may lead to prolonged allograft survival.
